# Case Report: A Case of Hailey–Hailey Disease Mimicking Condyloma Acuminatum and a Novel Splice-Site Mutation of ATP2C1 Gene

**DOI:** 10.3389/fgene.2021.777630

**Published:** 2021-12-14

**Authors:** Yuwei Dai, Lingling Yu, Yu Wang, Min Gao, Peiguang Wang

**Affiliations:** ^1^ Department of Dermatology, The First Affiliated Hospital, Anhui Medical University, Hefei, China; ^2^ Institute of Dermatology, Anhui Medical University, Hefei, China; ^3^ Key Laboratory of Dermatology, Anhui Medical University, Ministry of Education, Hefei, China; ^4^ Provincial Laboratory of Inflammatory and Immune Mediated Diseases, Hefei, China

**Keywords:** hailey–hailey disease, ATP2C1 gene, mutation, acantholytic dyskeratosis, familial benign chronic pemphigus

## Abstract

Hailey–Hailey disease (HHD) is a rare autosomal-dominant blistering disorder characterized by recurrent vesicular and erosive lesions at intertriginous sites. We described a 24-year-old male who presented with multiple bright red verrucous papules in his mons pubis, bilateral groins, scrotum, perineum, and crissum, clinically resembling condyloma acuminatum. The histopathology showed extensive acantholysis with the characteristic appearance of a dilapidated brick-wall. The mutation analysis revealed a novel splice-site mutation in the *ATP2C1* gene. The patient was definitely diagnosed with HHD. The antibacterial treatments resulted in a dramatic improvement. Our findings help to broaden the understanding of clinical manifestations of HHD and improve the clinical diagnosis and treatment of this disease.

## Introduction

Hailey–Hailey disease (HHD), also known as familial benign chronic pemphigus, is a rare autosomal-dominant blistering disease with an estimated incidence of approximately 1/50,000 (Ben Lagha et al., 2020). It is characterized by recurrent vesicles, erosions, and macerated plaques involving the intertriginous areas, such as the lateral neck, axillae, groins, and perianal areas. The disease usually gives rise to severe discomfort and chronic relapse, so greatly impacts a patient’s quality of life. The affected individuals are usually presented with clinical findings between the third and fourth decades of life. HHD is caused by mutations in the *ATP2C1* gene on chromosome 3q21 encoding the human secretory pathway Ca^2+^/Mn^2+^ ATPase isoform 1 (hSPCA1) in the Golgi apparatus. hSPCA1, a calcium transporter protein, regulates the concentration of both Ca^2+^ and Mn^2+^ in the Golgi complex (Hu et al., 2000; Sudbrak et al., 2000). The intracellular Ca^2+^ stores play a pivotal role in maintaining epidermal integrity. The loss-of-function mutation in the *ATP2C1* gene leads to defective calcium homeostasis, loss of cell–cell adhesion of keratinocytes, and acantholysis (Fairclough et al., 2003; Vanoevelen et al., 2007). We report a 24-year-old male, who was presented with condyloma acuminatum-like lesions and a novel splice-site mutation in the *ATP2C1* gene from a Chinese family with HHD.

## Case Presentation

**FIGURE 1:**
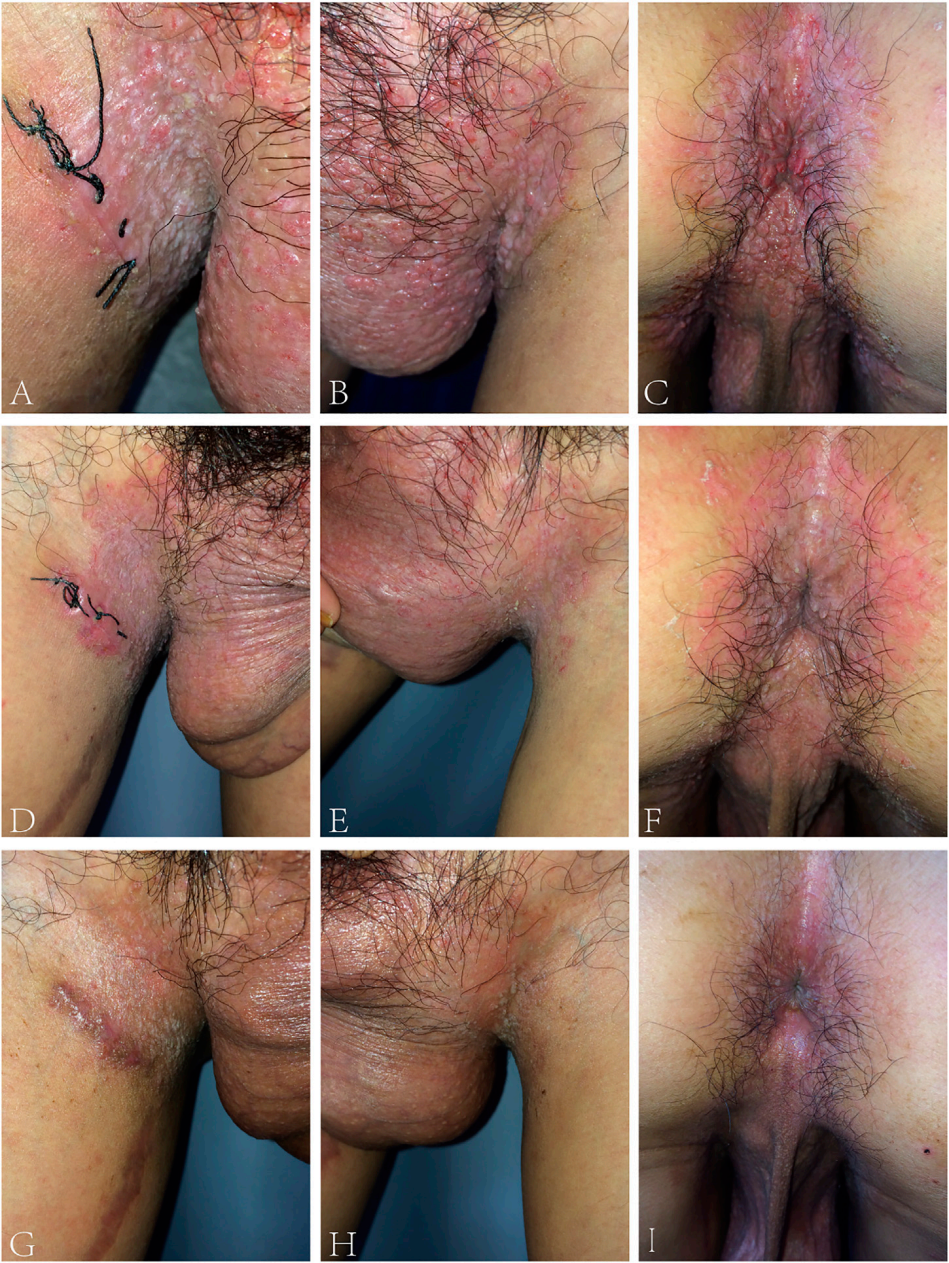
**(A–C)** Bright red warty papules on bilateral groins, scrotum, perineum, and crissum of the proband. **(D–F)** Almost all of the warty papules subsided after 5 days of treatment. **(G–I)** 4 weeks later, only a few greyish white papules remained.

**FIGURE 2:**
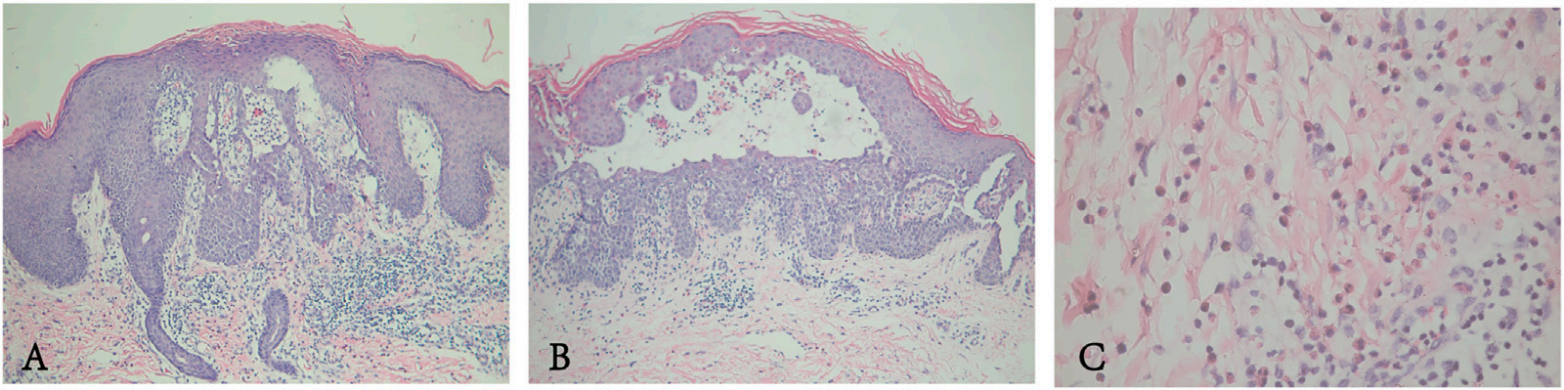
**(A,B)** Epidermal hyperkeratosis accompanied by parakeratosis, acantholysis with the appearance of a dilapidated brick-wall, and formation of intraepidermal blisters. **(C)** Some lymphocytes and a few eosinophils in the dermis.

The proband was a 24-year-old male, who presented with pruritic skin lesions in his genital and perianal regions for more than 7 years. On physical examination, multiple bright red verrucous papules were observed in his mons pubis, bilateral groins, scrotum, perineum, and crissum (Figures 1A–C). His general health was normal. Mycological examination of scales showed no hyphae and spores under a light microscope. The acetic acid white test was negative. PCR detection of the HPV DNA showed the absence of HPV. All blood TRUST, TPPA, and anti-HIV antibody tests were also negative. Histopathology of a biopsy from his right groin showed epidermal hyperkeratosis, parakeratosis, downward proliferation with a finger-like protrusion, and acantholysis with the appearance of a dilapidated brick-wall as well as the formation of a blister in the epidermis. In addition, there were vascular dilatation in the dermal papilla and infiltration of lymphocytes and eosinophils in the dermis (Figure 2). His mother was a 45-year-old woman, who presented with relapsing flares of mild erythema under her armpits for many years. His father was unaffected. The proband was diagnosed with Hailey–Hailey disease on the basis of his clinical and laboratory findings. He was administered with the treatment of oral cephradine, cleansing of 1:5,000 potassium permanganate solution, and topical 2% mupirocin ointment. The warty papules were dramatically improved after 5 days of his second visit (Figures 1D–F). Oral cetirizine and cyproheptadine were then given to relieve severe itching. Four weeks later, a few greyish white small papules were still present in his bilateral groins (Figures 1G–I). Therefore, the combination of tacalcitol ointment and mucopolysaccharide polysulfate cream was then used.

## Materials and Methods

The peripheral blood of the proband and his parents was collected after obtaining their informed consent and the approval of the Ethics Committee of Anhui Medical University. Genomic DNA was extracted by the DNA extraction kit (Promega, Madison, WI, United States).

Primer Premier 5.0 (Primer Biosystems, Foster City, CA, United States, Resource Identification Portal, RRID: SCR_004098) was used to design primers of all exons of *ATP2C1*. The PCR products of genomic DNA were then sequenced by using an ABI 3730xl DNA analyzer (ABI, Foster City, CA, United States, USEDit, RRID: SCR_018018), and the nucleotide sequences were analyzed by FinchTV (Version 1.4).

The variant was annotated against NCBI RefSeq: NM_001001486.1 and checked for the presence in ClinVar,^1^ ExAC, 1000G,^2^ and *ATP2C1* LOVD v.3.0 databases^3^.

## Result

**FIGURE 3:**
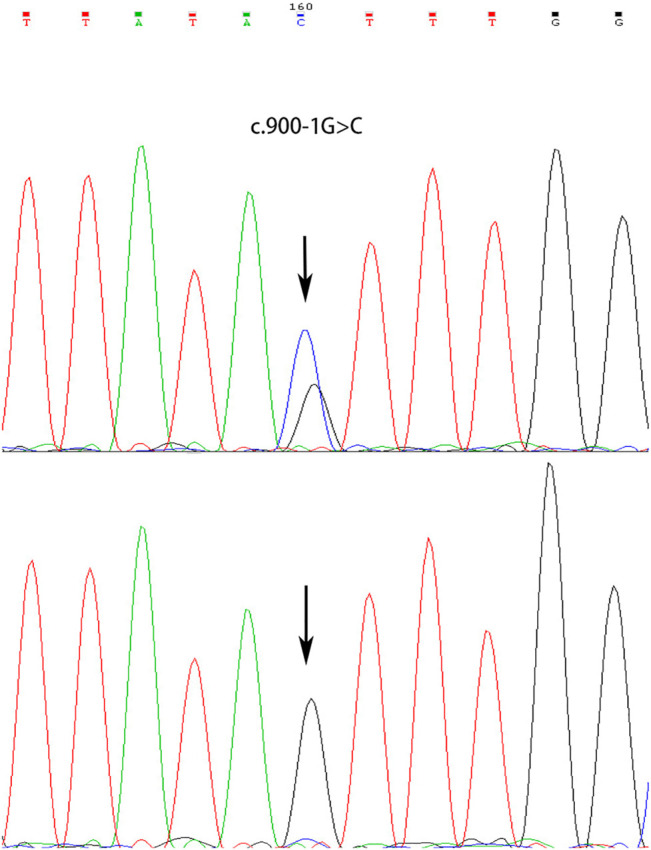
Heterozygous splicing mutation in *ATP2C1* identified in the proband and his mother **(upper)** and sequencing result of his father **(normal, lower)**.

A novel heterozygous splice-site mutation c.900-1G > C in the *ATP2C1* gene was identified in both proband and his mother, whereas his father showed a wild-type sequence (Figure 3). The mutation was predicted to be “disease-causing” in MutationTaster^4^ and “Alteration of the WT acceptor site, most probably affecting splicing” in Human Splicing Finder^5^. The genotype is perfectly co-segregated with the clinical phenotype in this family. The finding of gene mutation analysis provides strong evidence to support the diagnosis of HHD.

## Discussion

Typically, the patients with HHD present with flaccid vesicopustules, crusted erosions, macerations, or fissures in the friction-prone skin folds. However, the vulva, back, or inframammary areas were also affected (Vasudevan et al., 2015; Reyes et al., 2016; Lemieux and Funaro, 2020; Sousa Gomes et al., 2020). Rarely, mucosal involvement was observed including conjunctival, oral, esophageal, and vaginal mucosa (Burge, 1992; Oğuz et al., 1997; Fresco et al., 2020). There are some clinical variants in this disease, such as generalized, segmental, vesiculobullous, condylomatous, circinate or annular, lichenoid, and psoriasiform HHD (Hwang et al., 2003; Vilmer and Dehen, 2004; Ghosh et al., 2017; Plaza et al., 2017; Ni et al., 2018; Leducq et al., 2020; Ting et al., 2021). HHD was concomitant with bullous pemphigoid, eczema herpeticum, and human papillomavirus infection in a few cases (Chan et al., 2007; Shah et al., 2020; Li et al., 2021).

In our study, the proband presented with multiple bright red verrucous papules, clinically resembling condyloma acuminatum. Condyloma acuminatum is a benign proliferative disease of mucocutaneous tissues caused by the infection of human papillomavirus (HPV). Its typical feature is red corolliform or cauliflower-like papules or plaques on the anogenital areas. Usually, histopathological examination demonstrates epidermal hyperkeratosis and koilocytes in the granular and upper spinous layers (Chan, 2019). The diagnosis of condyloma acuminatum can be easily excluded according to his histopathological finding and absence of HPV DNA for the proband.

So far, a total of 250 public pathogenic variants in the *ATP2C1* gene have been described in the *ATP2C1* LOVD v.3.0 database (Accessed on Nov 21, 2021). There are 16.8% variants occurring in the splice region and 29.6% causing frameshift mutations, 25.2% causing missense mutations, 6.8% causing in-frame deletions, 0.4% causing no protein production, 0.4% causing in-frame indels, 22.8% causing stop changes, and 14.8% are unknown. No significant associations between the genotype and phenotype have been found. The mutation identified in our study is located at the acceptor splice site of intron 11 that probably affects the complete splicing of exon 12. Exon 12 of *ATP2C1* encodes the location of a protein associated with calcium binding (Deng and Xiao, 2017). The mutation c.900-1G > C in the ATP2C1 gene is previously not described.

HHD is one of the acantholytic conditions or papular acantholytic dyskeratosis. The common histopathological findings are the epidermal parakeratosis, dyskeratosis, suprabasal acantholytic cleft or bulla, and the typical appearance of “dilapidated brick-wall.” In general, intercellular deposition of IgG and complement 3 (C3) is not detected in the epidermis of HHD patients in contrast to autoimmune pemphigus. However, one HHD patient had linear deposition of C3 along the dermoepidermal junction (Gu et al., 1999). Anti-desmoglein and anti-desmocollin antibodies are found in sera of two cases of HHD patients (Bennani et al., 2012; Ueo et al., 2015). Moreover, fixed and soluble immune complexes are present in the epidermis of patients (Makhneva and Beletskaya, 2007). Regretfully, we did not perform direct immunofluoresence staining and serum autoantibodies detection for the patient. Probably, the formation of anti-desmoglein antibodies, anti-desmocollin antibodies, and immune complexes is associated with the unmasking of desmosomal antigens due to acantholysis. These conditions suggest that immunological factors are also involved in the pathogenesis of HHD in addition to a genetic defect. The speculation could provide a plausible explanation for the use of corticosteroids or immunosuppressants in HHD. In addition, abnormally elevated oxidative stress levels have been found in the keratinocytes of HHD; a small number of patients with refractory symptoms achieved good efficacy with antioxidant drugs (Biolcati et al., 2014).

There are a variety of triggering factors aggravating HHD, such as ultraviolet exposure, skin infection, high temperature, sweating, friction, trauma, menstruation, and pregnancy (Engin et al., 2015). So, these unfavorable factors should be avoided or eliminated. At present, there is no known cure for HHD. Multiple therapeutic options have been reported. Conventional treatments include topical antibacterial or antifungal agents, oral antibiotics, moderate to potent topical corticosteroids, topical tacrolimus ointment, and topical vitamin D3 analogs. Although ultraviolet light may exacerbate HHD, some patients respond well to narrow-band UVB phototherapy (Mizuno et al., 2014; Abaca et al., 2018). Systemic corticosteroids, cyclosporin, methotrexate, acitretin, or alitretinoin may be considered for generalized HHD (Sárdy and Ruzicka, 2014; Ben Lagha et al., 2020); however, long-term use is not recommended because of serious side effects. Multiple new treatments have been demonstrated to be effective in some refractory cases of HHD in recent years, including botulinum toxin, naltrexone, dupilumab, apremilast, photodynamic therapy, common or fractional CO_2_ laser, 595-nm pulsed dye laser, and electron beam radiotherapy (Di Altobrando et al., 2020; Michael et al., 2020; Alzahrani et al., 2021; Dulmage et al., 2021; Zhang et al., 2021). Long-term improvement was observed in some patients treated with various laser ablation or electron beam radiotherapy (Leung et al., 2018).

In conclusion, we provided one case of HHD with a rare clinical feature and a novel splice-site mutation in the *ATP2C1* gene. Multiple warty papules dramatically resolved after antibacterial treatment. Our findings help to broaden the understanding of the clinical of HHD and improve the clinical diagnosis and treatment of this disease.

## Data Availability

The datasets for this article are not publicly available due to concerns regarding participant/patient anonymity. Requests to access the datasets should be directed to the corresponding author.

## Footnotes

1
http://www.ncbi.nlm.nih.gov/clinvar.

2
https://www.internationalgenome.org/.

3
http://lovd.nl/ATP2C1.

4
http://www.mutationtaster.org/.

5
http://www.umd.be/HSF3/.
